# Supplementary material for: Transient expression of an adenine base editor corrects the Hutchinson-Gilford progeria syndrome mutation and improves the skin phenotype in mice
Source: Nat Commun. 2022 Jun 2;13:3068. doi: 10.1038/s41467-022-30800-y (PMC9163128; doi:10.1038/s41467-022-30800-y)
Supplement: Supplementary file 1 — Supplementary info [file 41467_2022_30800_MOESM1_ESM.pdf]

Supplementary Information: Transient expression of an adenine base editor corrects the Hutchinson-Gilford progeria syndrome mutation and improves the skin phenotype in mice

Daniel Whisenant[1,#], Kayeong Lim[2,#], Gwladys Revêchon[1], Haidong Yao[1], Martin O. Bergo[1], Piotr Machtel[1], Jin-Soo Kim[2], Maria Eriksson[1,*]

This file includes

Supplementary Figs. 1-7

Supplementary Table 1

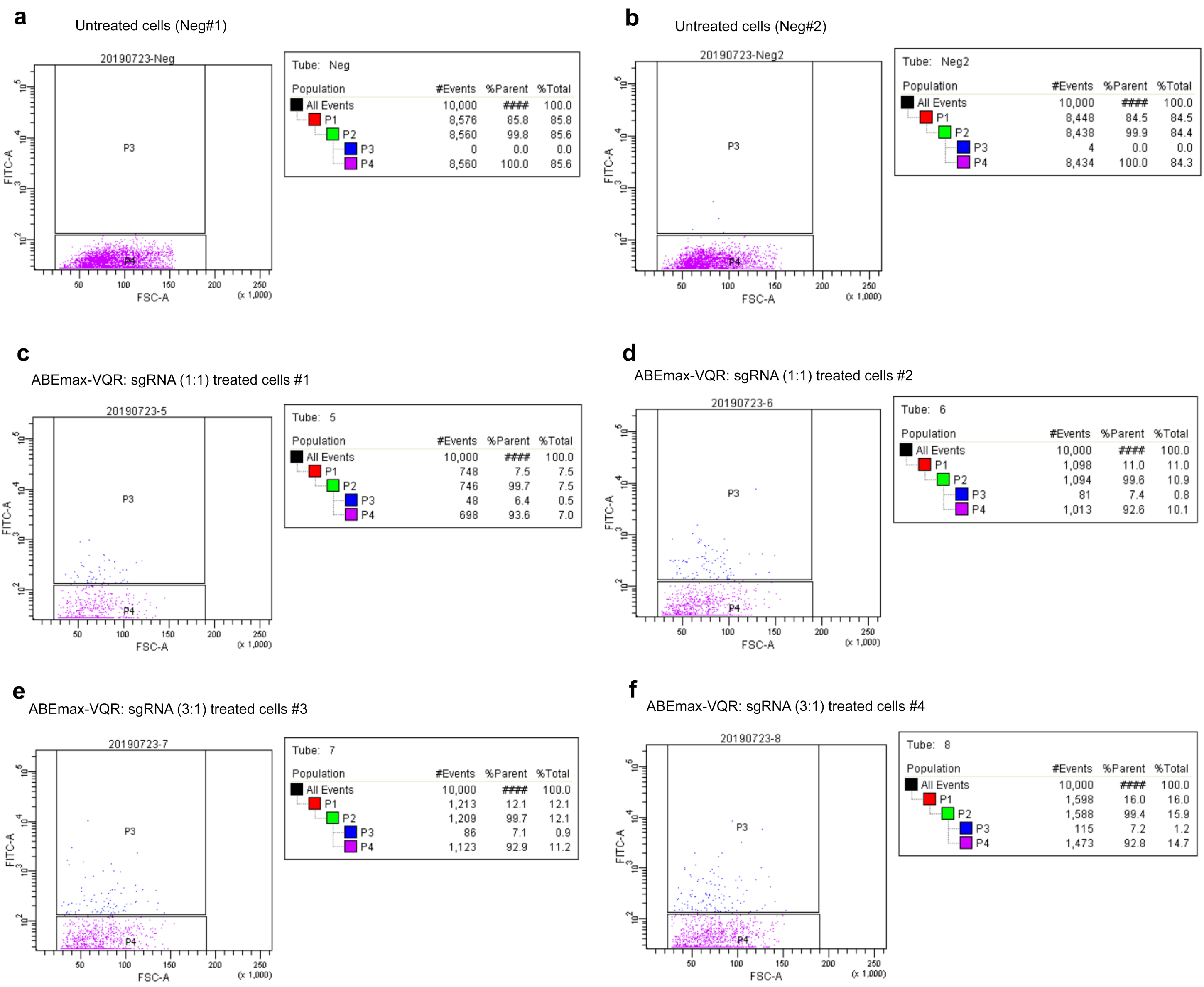

Supplementary Fig. 1: FACS data for ABE mediated correction of the pathogenic HGPS mutation in patient cells. (a) FACS plots of untransfected HGPS patient derived B-LCL cells (Neg#1) have no GFP signal (P3 gate). **(b)** FACS plots of untransfected HGPS patient derived B-LCL cells (Neg#2) have no GFP signal (P3 gate). **(c)** FACS plots of transfected HGPS patient derived B-LCL (ABEmax-VQR: sgRNA (1:1) treated cells #1) cells with a 1:1 ABEmax-VQR: sgRNA plasmid ratio show GFP signal (P3) for enrichment of cells that express the ABEmax-VQR and sgRNA plasmids. **(d)** FACS plots of transfected HGPS patient derived B-LCL (ABEmax-VQR: sgRNA (1:1) treated cells #2) cells with a 1:1 ABEmax-VQR: sgRNA plasmid ratio show GFP signal (P3) for enrichment of cells that express the ABEmax-VQR and sgRNA plasmids. **(e)** FACS plots of transfected HGPS patient derived B-LCL (ABEmax-VQR: sgRNA (3:1) treated cells #3) cells with a 1:1 ABEmax-VQR: sgRNA plasmid ratio show GFP signal (P3) for enrichment of cells that express the ABEmax-VQR and sgRNA plasmids. **(f)** FACS plots of transfected HGPS patient derived B-LCL (ABEmax-VQR: sgRNA (3:1) treated cells #4) cells with a 1:1 ABEmax-VQR: sgRNA plasmid ratio show GFP signal (P3) for enrichment of cells that express the ABEmax-VQR and sgRNA plasmids.

a

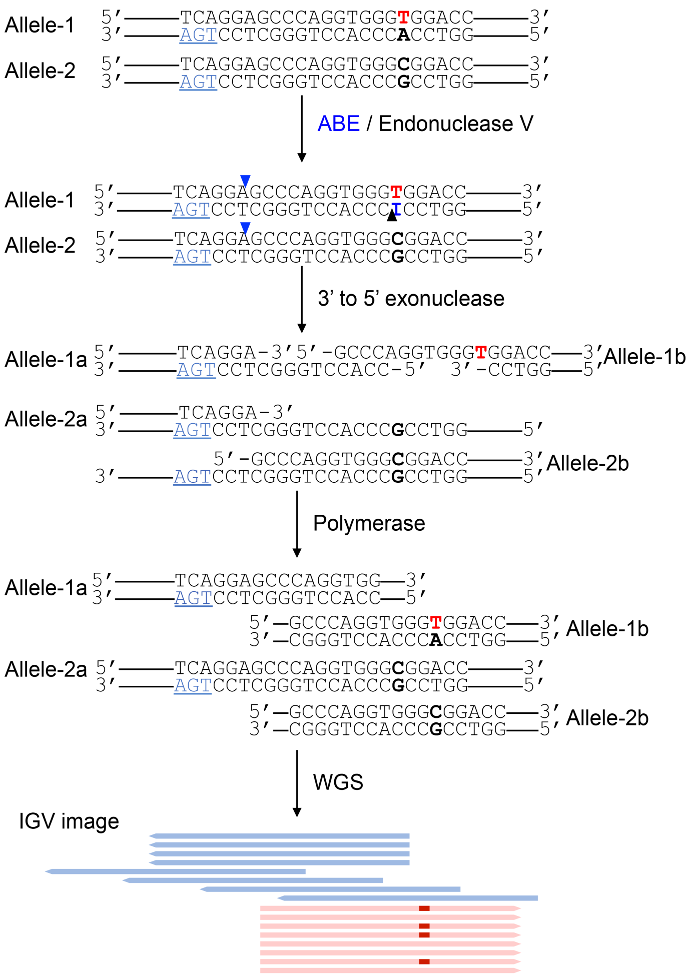

b

ON/Off-target(OT) loci	Chromosomal location	Sequence reference
ON	chr1:156138596	GGTCCACCCACCTGGGCTCCTGA
OT1	chr14:19159884	taggtACCCACCTGGGCTCCCGT
OT2	chr9:123431435	GGTCCACCCtCCTGGGaTCCTGA
OT3	chr17:15822095	cagCCACCCACCTGGGCTCCTGA
OT4	chr9:63710987	taggtACCCACCTGGGCTCCAGT
OT5	chr16:87936978	tGTCCACCCACCaGGGCTCCTGA
OT6	chr7:151434192	GGTCC-CCCACCTGGGgTCCCGA
OT7	chr1:64384497	No homology
OT8	chr9:137435889	GaTCCAcCCCACCcGGGCTCCCAG
OT9	chr18:2745186	No homology
OT10	chr11:1532499	aacagcCCCACCTGGGCTCCTGA
OT11	chr20:44765635	tGaCCACCCACCcaGGCTCCTGA
OT12	chr22:27674162	tGTCCA-CCACCTGGGCTCCCGT
OT13	chr21:9037310	taggtAtCCACCTGGGCTCCGGT
OT14	chr21:10126387	taggtACCCACCTGGGCTCCAGT
OT15	chr16:84531390	GaTCCaCCCACCTGaGGCTCCTGA
OT16	chr2:131757385	taggtACCCACCTGGGCTCCGGT
OT17	chr6:31553908	GaTCCACCCACCtTGGGCTCCCAG
OT18	chr9:40113113	taggtACCCACCTGGGCTCCAGT
OT19	chr10:97169677	ccagagCCCACCTGGGCTCCCTG
OT20	chr1:44683346	GGgCCtCCCACCTGaGCTCCAGA
OT21	chr4:142139924	GaTCCACCCACCTGGGCTtCCAA
OT22	chr20:46383274	aagCCACCCtCCTGGGCTCCAGA
OT23	chr15:90226584	GaTCCACCCACCtTGGGCTCCCAG
OT24	chr18:79187284	tGaCCACCCACCTGGGCTaCTGT
OT25	chr3:23701424	GaTCCACCCACCtTGGGCTCCCGA
OT26	chr17:20639978	cagCCACCCACCTGGGCTCCTGA
OT27	chr2:113639208	ccTCCA-CCcCCTGGGCTCCAGT
OT28	chr11:4071435	ccTCCA-CCcCCTGGGCTCAAGA
OT29	chr15:72439904	tttttcCCCACCTGGGCTCCCGC
OT30	chr12:119636095	GtcCCACCC-CCTGGaCTCCAGG
OT31	chr1:9841962	tGTagACCCACCTGGGCTCCTGG
OT32	chr17:81530475	tcaCtACCCACCTGGGCTCCTGG
OT33	chr1:156061117	tcTCCACCCACCTGGGCcCCAGG
OT34	chr1:24964132	GGaCtcCCCACCTGGGCTCaGGA
OT35	chr1:154904123	GaTCCtCCCACCTGaGCTCCCAA
OT36	chr15:40908761	GaTCCtCCCACCcCaGGCTCCTGA
OT37	chr3:28238931	No homology
OT38	chr12:105471949	No homology
OT39	chr18:9064907	GaTCCACCCACCTtGGGCTCCCAA
OT40	chr15:29133368	GGcCCACCC-CCaGGGCTCCTGA
OT41	chr17:34394307	acTCCACCCACCTGGGCaaCAGA
OT42	chr3:109374902	GATCCgCCCACCTGGGCctCCGA

c

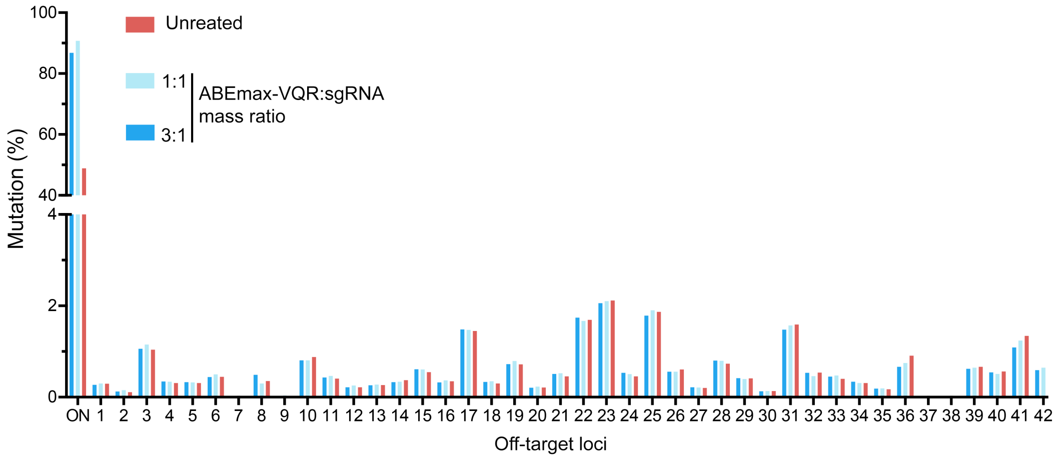

d

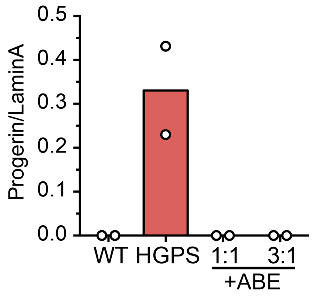

Supplementary Fig. 2: Identification of off-target loci using Digenome-seq and validation by targeted deep sequencing. (a) Overview of Digenome-seq with c.1824C>T targeting ABE-VQR from genomic DNA of HGPS patient derived B-lymphoblasts carrying the mono allelic *LMNA* c.1824C>T mutation. **(b)** Off-target candidate sites identified by Digenome-seq. mismatched bases, PAM sequences, and a DNA bulge are shown in red, blue, and green, respectivley. A RNA bulge is represented as a dash. **(c)** Base editing efficiencies were measured using targeted deep sequencing at 42 candidate off-target sites in DNA of HGPS patient derived B-lymphoblasts, transfected with the ABEmax-VQR: sgRNA plasmid mass ratio of 1:1 and 3:1. **(d)** Western blot analysis of progerin abundance of WT and HGPS patient derived B-lymphoblasts, transfected with the ABEmax-VQR: sgRNA plasmid mass ratio of 1:1 and 3:1. Data are obtained from n=2 independent experiments and are represented as mean. For **(d)** data are available as Source Data file.

a

ON/Off-target(OT) loci	Chromosomal location	Sequence
ON	chr1:156138596	GGTCCACCCACCTGGGCTCCTGA
OT1	chr1:219489120	GaTCCACCCACCTGGGCcCCTGA
OT2	chr1:227794622	GGcCCACCCACCTcGGCTCCCTG
OT3	chr14:21226636	GGTCCACCCgCCTGGGCTCaAGG
OT4	chr14:77324384	GaTCCACCCACCTtGGCTCCCGA
OT5	chr15:72528770	GcTCCACCCtCCTGGGCTCCAGC
OT6	chr16:7096451	GGTCCACCCACCaGGGCTgCTGT
OT7	chr16:30335860	tGTCCACCCACCaGGGCTCCTGA
OT8	chr16:47009950	GaTCCACCCACCTtGGCTCCCGA
OT9	chr16:87936967	tGTCCACCCACCaGGGCTCCTGA
OT10	chr16:88421166	GGTgCcCCCACCTGGGCTCCTGC
OT11	chr2:23715331	GaTCCACCCACCTGGcCTCCCGA
OT12	chr2:200977607	GaTCCACCCACCTGGcCTCCCAG
OT13	chr3:33384318	GGTCCACCCACCTtGGCTtCTGA
OT14	chr5:54626394	GGTCCtCCCACCTtGGCTCCCAG
OT15	chr5:55287197	GaTCCACCCACCTcGGCTCCCAG
OT16	chr6:146173145	GaTCCACCCACtTGGGCTCCCAG
OT17	chr8:141912343	aGTCCAgCCACCTGGGCTCCCTG
OT18	chr9:123431427	GGTCCACCCtCCTGGGaTCCTGA
OT19	chr9:127091278	GGgCCACaCACCTGGGCTCCAGT

b

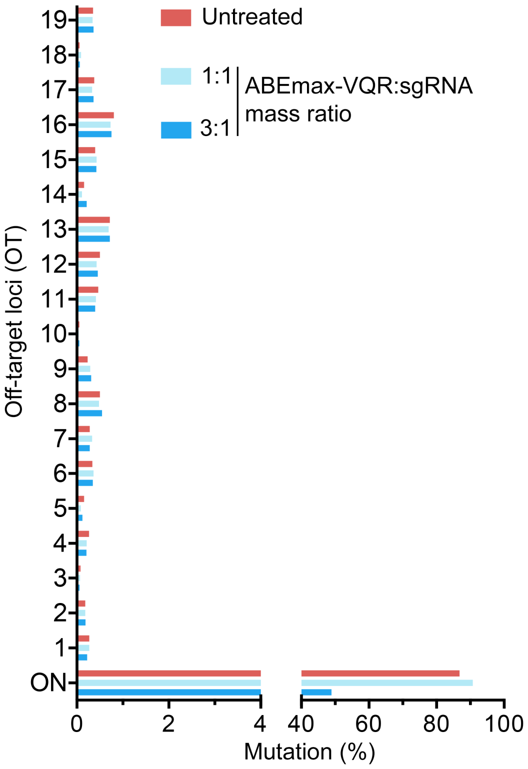

Supplementary Fig. 3: Cas-Offinder analysis of potential off-target loci.

(a) Identification of 19 potential off-target loci with the Cas-offinder algorithm across the human genome (GRCh38/hg38). Mismatched bases and PAM sequences are shown in red and blue, respectivley. **(b)** Base editing efficiency measured by targeted deep sequencing at 20 potential off-tagret loci does not show an increase in the mutation frequency *in vitro*. HGPS patient derived lymphoblasts were treated with the ABEmax-VQR to sgRNA plasmid mass ratio of 1:1 and 3:1. Values are obtained from 2 biologically independent replicates in each group and represented as mean.

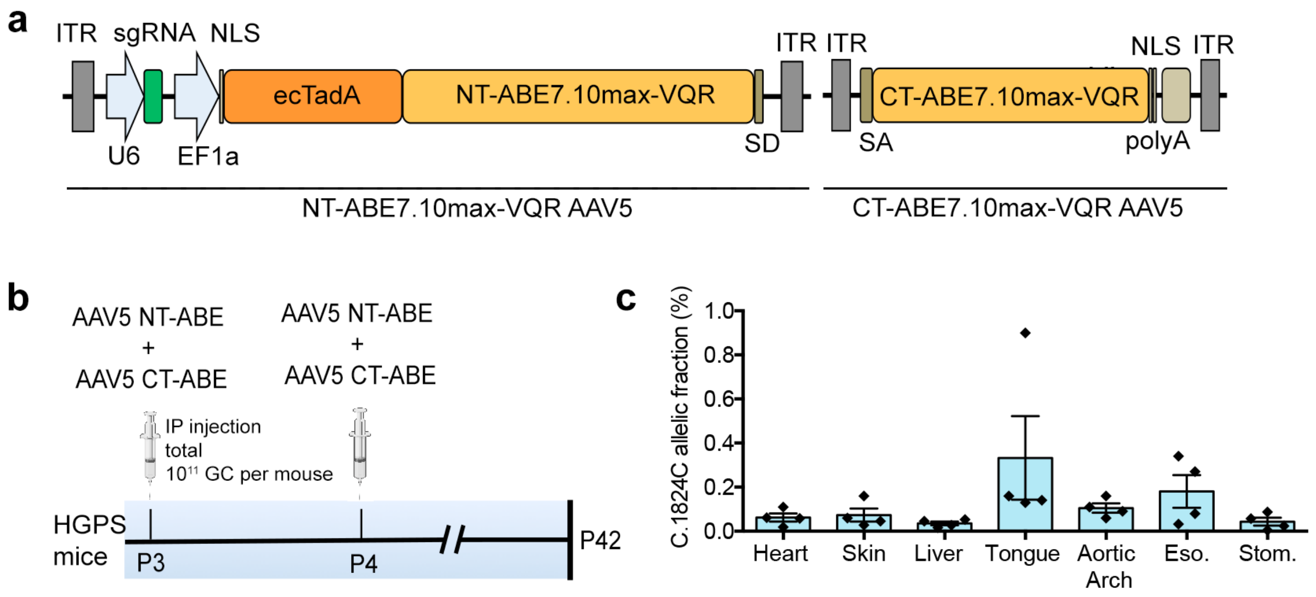

Supplementary Fig. 4: Systemic AAV5 mediated ABE treatment of HGPS mice demonstrates low c.1824T mutation editing. (a) AAV5 split N-terminal (NT) and C-terminal (CT) ABEmax-VQR encoding vectors. Each NT-ABE splice donor and CT-ABE splice acceptor vector is encoded in a separate AAV5 particle. If both NT-ABE and CT-ABE vectors transfect the same cell the functional ABE is generated **(b)** Systemic intraperitoneal (IP) injection of HGPS mice at P3 and P4 with 10^{11}GC of AAV5 NT-ABE and CT-ABE virus, tissues were collected at P42. **(c)** Fractional abundance of the c.1824C WT allele at P42 in AAV5-ABE treated mice shows a low editing efficiency in heart, skin, liver, tongue, aortic arch, esophagus and stomach tissue (n=4 biologically independent samples). Data are represented as mean +/- SEM and are provided as Source Data file.

a

ddPCR-RED

Sample	ABE#1	ABE#1 +PCR	ABE#2	ABE#2 +PCR
Reference allele	WT (c.1824C)	WT (c.1824C)	WT (c.1824C)	WT (c.1824C)
Reference allele fractional abundance	43,80%	0,00%	19,30%	0,00%

MiSeq.

Sample	ABE#1	ABE#2
Reference allele	WT (c.1824C)	WT (c.1824C)
Reference allele count	783 (2,26%)	530 (1,69%)

b

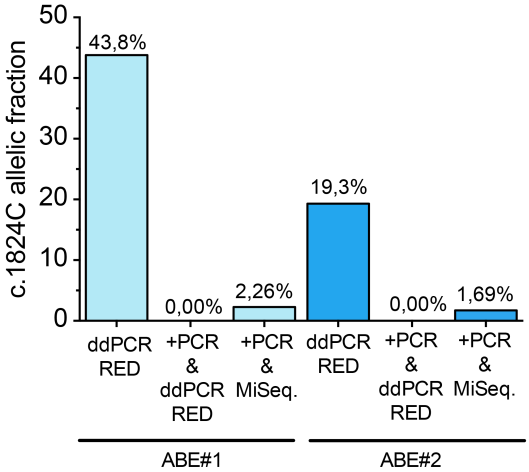

c

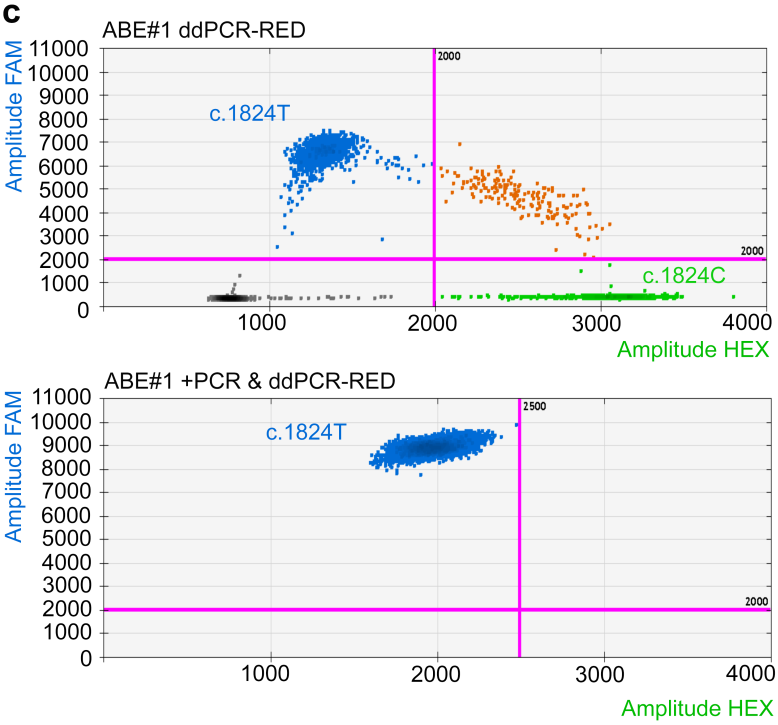

Supplementary Fig. 5: Rare event detection droplet digital PCR and targeted amplicon sequencing of LF-ABE treated skin, two days post treatment. (a) DdPCR-RED directly after DNA isolations, two days post LF-ABE treatment shows a c.1824C allele fraction of 43,8% (ABE#1) and 19,3% (ABE#2). After LMNART10F/R primer amplification (+PCR) the c.1824C allelic fraction is not detected anymore. Targeted amplicon sequencing (MiSeq.) after LMNART10F/R primer amplification shows a lower c.1824C allelic fraction of 2,26% (ABE#1) and 1,69% (ABE#2) in comparison to the ddPCR-RED assay before the PCR primer amplification. **(b)** Overview of the ddPCR-RED assay before (ddPCR-RED) and after primer amplification (+PCR & ddPCR-RED) in comparison to targeted amplicon sequencing (+PCR & MiSeq.). **(c)** Example ddPCR-RED 2D plot before (ABE#1 ddPCR-RED) and after primer amplification (ABE#1 +PCR & ddPCR-RED), blue dots (c.1824T alleles), green dots (c.1824C alleles), orange dots (c.1824T and c.1824C alleles). For **(b)** data are represented as mean.

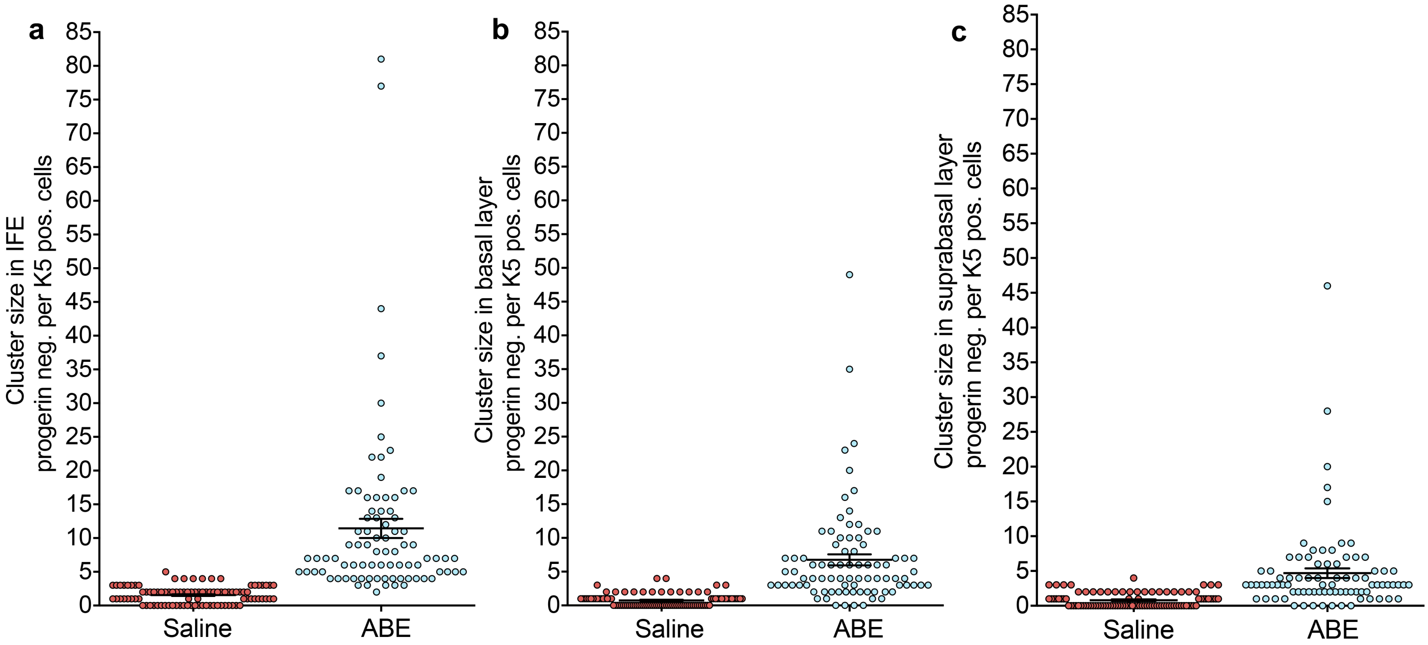

Supplementary Fig. 6: Distribution of progerin negative cell clusters in saline and LF-ABE treated skin samples at 4 weeks post treatment. (a) Number of progerin negative per Keratin-5 (K5) positive cells/cluster in the IFE, basal layer **(b)** and suprabasal layer **(c)** of saline treated HGPS mice (n=5, biologically independent samples) and LF-ABE treated HGPS mice (n=5 biologically independent samples). Data are represented as mean +/- SEM and are provided as Source Data file.

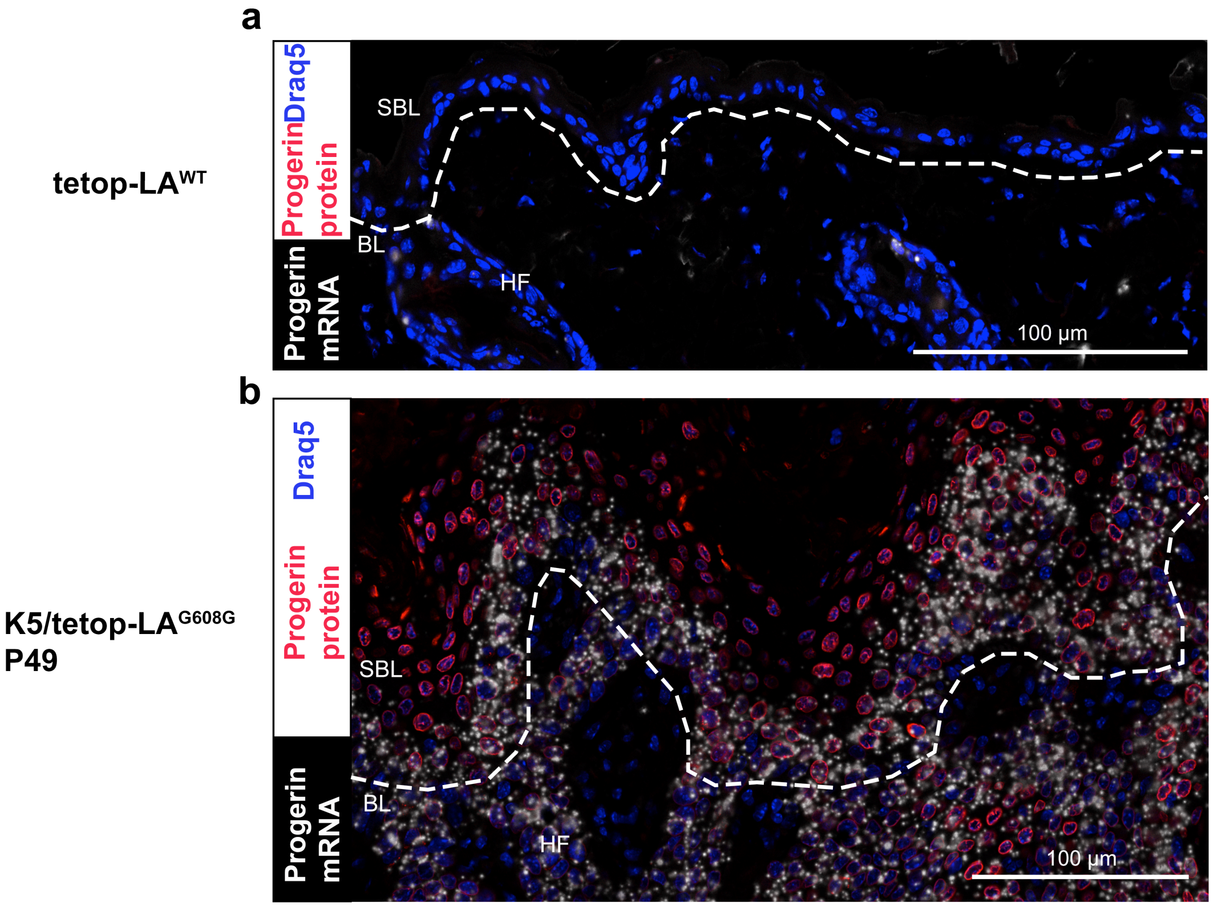

Supplementary Fig. 7: Specificity of progerin in situ hybridization and progerin protein staining in mouse skin. (a) No detection of progerin mRNA (white) and protein staining (red) in tetop-LA^{WT} control mouse skin that does not express progerin in the basal (BL) and suprabasal layer (SBL) or hair follicle (HF). **(b)** In K5/tetop-LA^{G608G} mouse skin we could detect progerin mRNA and protein expression in the basal layer as well as in the hair follicle. In the suprabasal layer we could detect only progerin protein staining in agreement that progerin transcript expression is limited to K5 positive cells residing in the basal layer and hair follicle. Scale bar: a, b = 100 µm.

Supplementary Table 1. ***In silico*** **alternative splicing prediction of the** ***LMNA*** **exon 11 cryptic splice site using four different models.** The A10 (c.1820 A:T to G:C) and the A6 (c.1824 A:T to G:C) nucleotide transitions are marked in red.

**Sequence *LMNA* exon 11**	**Mutation**	**Maximum Entrophy Model (MAXENT)**	**Maximum Dependence Decomposition Model (MDD)**	**First-order Markov Model (MM)**	**Weight Matrix Model (WMM)**
cagGTGGGC	WT Reference	8.07	11.98	6.97	8.10
cagGTGGGT	c.1824C>T (p.G608G)	8.56	13.38	8.54	9.32
cagGCGGGC	c.1820T>C (p.V607A)	0.31	4.22	-0.78	0.34
cagGCGGGT	c.1824C>T (p.G608G) & c.1820T>C (p.V607A)	0.81	5.62	0.79	1.57
