# Supplementary material for: Transient expression of an adenine base editor corrects the Hutchinson-Gilford progeria syndrome mutation and improves the skin phenotype in mice
Source: Nat Commun. 2022 Jun 2;13:3068. doi: 10.1038/s41467-022-30800-y (PMC9163128; doi:10.1038/s41467-022-30800-y)
Supplement: Supplementary file 2 — Description of additional supplementary files [file 41467_2022_30800_MOESM2_ESM.docx]

**Description of Additional Supplementary Files**

**File name:**

Supplementary Data 1

**Description:**

Primer sequences for targeted deep sequencing of the c. 1824C>T on-target site. PCR primers for validation at 42 digenome-positive off-target sites. PCR primers for validation at 19 predicted off-target sites using Cas-Offinder.
