# Supplementary material for: Transient expression of an adenine base editor corrects the Hutchinson-Gilford progeria syndrome mutation and improves the skin phenotype in mice
Source: Nat Commun. 2022 Jun 2;13:3068. doi: 10.1038/s41467-022-30800-y (PMC9163128; doi:10.1038/s41467-022-30800-y)
Supplement: Supplementary file 4 — Reporting Summary [file 41467_2022_30800_MOESM4_ESM.pdf]

nature portfolio

Corresponding author(s): Maria Eriksson

Last updated by author(s): Jan 7, 2022

Reporting Summary

Nature Portfolio wishes to improve the reproducibility of the work that we publish. This form provides structure for consistency and transparency in reporting. For further information on Nature Portfolio policies, see our Editorial Policies and the Editorial Policy Checklist.

Statistics

For all statistical analyses, confirm that the following items are present in the figure legend, table legend, main text, or Methods section.

n/a	Confirmed	
[ ]	[x]	The exact sample size (n) for each experimental group/condition, given as a discrete number and unit of measurement
[ ]	[x]	A statement on whether measurements were taken from distinct samples or whether the same sample was measured repeatedly
[ ]	[x]	The statistical test(s) used AND whether they are one- or two-sided<br>*Only common tests should be described solely by name; describe more complex techniques in the Methods section.*
[x]	[ ]	A description of all covariates tested
[ ]	[x]	A description of any assumptions or corrections, such as tests of normality and adjustment for multiple comparisons
[ ]	[x]	A full description of the statistical parameters including central tendency (e.g. means) or other basic estimates (e.g. regression coefficient) AND variation (e.g. standard deviation) or associated estimates of uncertainty (e.g. confidence intervals)
[ ]	[x]	For null hypothesis testing, the test statistic (e.g. F, t, r) with confidence intervals, effect sizes, degrees of freedom and P value noted<br>*Give P values as exact values whenever suitable.*
[x]	[ ]	For Bayesian analysis, information on the choice of priors and Markov chain Monte Carlo settings
[x]	[ ]	For hierarchical and complex designs, identification of the appropriate level for tests and full reporting of outcomes
[x]	[ ]	Estimates of effect sizes (e.g. Cohen's d, Pearson's r), indicating how they were calculated

Our web collection on statistics for biologists contains articles on many of the points above.

Software and code

Policy information about availability of computer code

Data collection	Whole genome sequencing for Digenome-seq was performed using the HiSeq X Ten system (Illumina) at Macrogen. Targeted deep sequencing was performed using the MiniSeq system (illumina). Amplicon sequencing was performed using the MiSeq system (Illumina)
Data analysis	WGS data for Digenome-seq was analyzed using Digenome program (https://github.com/chizksh/digenome-toolkit2). Substitution and indel frequencies from targeted deep sequencing data were calculated with the online tools BE-analyzer (http://www.rgenome.net/be-analyzer/) and Cas-analyzer (http://www.rgenome.net/cas-analyzer/) current versions, or with source code (https://github.com/ibs-cge/maund). GraphPad Prism and Excel were used to generate figures and tables. For amplicon sequencing data analysis the FASTQ files from wild-type and conditioned samples are aligned against reference construct using BWA-mem v0.7.17 algorithm. Once aligned, the PCR duplicate reads are removed from BAM files using Picard v2.10.3 tool MarkDuplicates. Obtained aligned BAM files are sorted and indexed with Samtools v1.12. The SNPs/Mutations are called using Freebayes v1.3.2.GraphPad Prism and Excel were used to generate figures and tables. For ddPCR Rare Event Detection and Absolute Transcript Quantification analysis raw data for each sample was analysed using the manufacturers software (QuantaSoft, version 1.6, Bio-Rad). Microscopy image quantification was performed using the NIS elements analsysis software (Nikon, v.5.3.04). Splicing was analysed using four different splice score models with the current version of the MaxEntScan splice analysing online tool (http://hollywood.mit.edu/burgelab/maxent/Xmaxentscan_scoreseq.html). GFP-positive HGPS patient derived B-LCL cells were collected using a BD FACSAria III sorter (BD Biosciences) with FACSDiva software (version 6.1.3).

For manuscripts utilizing custom algorithms or software that are central to the research but not yet described in published literature, software must be made available to editors and reviewers. We strongly encourage code deposition in a community repository (e.g. GitHub). See the Nature Portfolio guidelines for submitting code & software for further information.

Data

Policy information about availability of data

All manuscripts must include a data availability statement. This statement should provide the following information, where applicable:

- Accession codes, unique identifiers, or web links for publicly available datasets
- A description of any restrictions on data availability
- For clinical datasets or third party data, please ensure that the statement adheres to our policy

The data supporting the findings from this study are available within the manuscript and its supplementary information. The DNA sequencing data has been deposited in the National Center For Biotechnology Information (NCBI) Sequence Read Archive (SRA) database with the BioProject accession code PRJNA744017 (https://www.ncbi.nlm.nih.gov/bioproject/PRJNA744017). Source data are provided with this paper.

Field-specific reporting

Please select the one below that is the best fit for your research. If you are not sure, read the appropriate sections before making your selection.

☒ Life sciences ☐ Behavioural & social sciences ☐ Ecological, evolutionary & environmental sciences

For a reference copy of the document with all sections, see nature.com/documents/nr-reporting-summary-flat.pdf

Life sciences study design

All studies must disclose on these points even when the disclosure is negative.

Sample size	To reduce the sample size to a minimum based on ethical guidelines but to statistically compare biologically independent samples sample size was minimum n=3. Sample size was not determined by a statistical method since the sample size was not large (n=25 animals in total).
Data exclusions	no data or samples were excluded from the experiments
Replication	With measurements from individual biological replicates (minimum, n=3) . All attempts of replication were successful with the indicated biological replicates in the manuscript.
Randomization	Sample allocations were not randomized. This was not relevant since we could directly measure treatment effects by conversion of the targeted point mutation by droplet-digital PCR rare event detection and by targeted deep sequencing on DNA level.
Blinding	For data analysis the investigators were blinded to group allocations. For sample collection blinding was not possible since treated animals needed to be specifically marked based on regulations of the animal housing facility.

Reporting for specific materials, systems and methods

We require information from authors about some types of materials, experimental systems and methods used in many studies. Here, indicate whether each material, system or method listed is relevant to your study. If you are not sure if a list item applies to your research, read the appropriate section before selecting a response.

Materials & experimental systems

n/a	Involved in the study	
☐	☒	Antibodies
☐	☒	Eukaryotic cell lines
☒	☐	Palaeontology and archaeology
☐	☒	Animals and other organisms
☒	☐	Human research participants
☒	☐	Clinical data
☒	☐	Dual use research of concern

Methods

n/a	Involved in the study	
☒	☐	ChIP-seq
☐	☒	Flow cytometry
☒	☐	MRI-based neuroimaging

Antibodies

Antibodies used

Lamin A/C Mouse mAb (E-1) # 376248 Santa Cruz
β-Actin Mouse mAb, clone AC-74, #A2228 Sigma-Aldrich
Lamin A/C Mouse mAB, clone JoL2, #MAB3211, Chemicon
Progerin Mouse mAb, clone 13A4, #ALX-804-662-R200, Enzo Life Sciences

	53BP1 Rabbit pAb #36823, Abcam<br>anti-keratin 5 pAb #905501, BioLegend<br>p84 mAb, clone 5E10, #GTX70220, GeneTex<br>anti-keratin 15 pAb, 833904, BioLegend<br>Peroxidase AffiniPure Goat Anti-Mouse pAb, IgG (H+L), 115-035-062, JacksonImmunoResearch
Validation	Lamin A/C Mouse mAb (E-1) # 376248 Santa Cruz: PMID: # 35104452<br>Cited by: Kronenberg-Tenga, R., et al. 2021. A Lamin A/C variant causing striated muscle disease provides insights into filament organization. J Cell Sci. 134: jcs256156.<br><br>β-Actin Mouse mAb, clone AC-74, #A2228 Sigma-Aldrich<br>Cited by: Tina M B, et al. Molecular Biology of the Cell (2011)<br>β-Actin specifically controls cell growth, migration, and the G-actin pool<br><br>Lamin A/C Mouse mAB, clone JoL2, #MAB3211, Chemicon<br>Cited by: Koblan et al., Nature 2021, In vivo base editing rescues Hutchinson-Gilford progeria syndrome in mice. Jan;589 (7843):608-614. doi: 10.1038/s41586-020-03086-7<br><br>Progerin Mouse mAb, clone 13A4, #ALX-804-662-R200, Enzo Life Sciences<br>Cited by: Viceconte N, McKenna T, Eriksson M. Low levels of the reverse transactivator fail to induce target transgene expression in vascular smooth muscle cells. PLoS ONE. 2014;9:e104098<br><br>553BP1 Rabbit pAb #36823, Abcam<br>Cited by: Weyburne E & Bosco G Cancer-associated mutations in the condensin II subunit CAPH2 cause genomic instability through telomere dysfunction and anaphase chromosome bridges. J Cell Physiol 236:3579-3598 (2021)<br><br>anti-keratin 5 pAb #905501, BioLegend<br>Cited by: Hübner A, et al. JNK and PTEN cooperatively control the development of invasive adenocarcinoma of the prostate. PNAS, 109: 12046-12051, Jul 2012<br><br>p84 mAb, clone 5E10, #GTX70220, GeneTex<br>Cited by: HHsiao YW et al. Clin Transl Med 2022; 12 (1):e724 Disruption of the pentraxin 3/CD44 interaction as an efficient therapy for triple-negative breast cancers<br><br>anti-keratin 15 pAb, 833904, BioLegend<br>Cited by: Ding X, et al. 2016. Nat Commun. 7:13226<br><br>Peroxidase AffiniPure Goat Anti-Mouse IgG (H+L), 115-035-062, JacksonImmunoResearch<br>Cited by: Szilágyi SS et al., BMC Biol (2022). Competition between type I activin and BMP receptors for binding to ACVR2A regulates signaling to distinct Smad pathways

Eukaryotic cell lines

Policy information about cell lines

Cell line source(s)	WT and HGPS Human B-lymphoblast cell lines were obtained from the Coriell Biobank (WT: AG03507, HGPS: AG03506)
Authentication	WT: AG03507<br>Authentication: By STR profiling, and mutation specific PCR primer amplification for the LMNA c.1824C>T transition<br>Eriksson et al. Nature 423: 293 (2003)<br><br>HGPS: AG03506<br>Authentication: By STR profiling, and mutation specific PCR primer amplification for the LMNA c.1824C>T transition<br>Eriksson et al. Nature 423: 293 (2003)
Mycoplasma contamination	All cells are negative for micoplasma, Gram+ and Gram- bacteria as well as mold and yeast contamination
Commonly misidentified lines<br>(See ICLAC register)	no commonly misidentified cell lines were used in the study

Animals and other organisms

Policy information about studies involving animals; ARRIVE guidelines recommended for reporting animal research

Laboratory animals	All animal experiments were performed in accordance with the Karolinska institutional guidelines and regulations. All procedures were approved by the Stockholm South Ethical review board (Dnr. 06088-2020). For animal experiment, heterozygous tetop-

	LAG608G (Eriksson et al., 2003; Sagelius et al., 2008) mice were intercrossed with heterozygous K5-tTA mice (Diamondet al., 2000) on the FVB/N strain. Experiments with WT and HGPS mice were performed on both genders. Mice were injected at 21 & 22 days after birth (P21, P22) or 47 & 48 days after birth (P47, P48) and sacrificed for analysis at either 23 days after birth (P23) or 49 days after birth (P49) control animals were age matched. Animals that appeared unhealthy before the start of the experiments were excluded. Except from genotype, no inclusion criterion was used. Animals were housed with a 12 hour light/dark cycle at 20-22°C, 50%-60% humidity in a pathogen-free animal facility at the Karolinska University Hospital with free access to water and food.
Wild animals	no wild animals were used in the study
Field-collected samples	no field collected samples were used in the study
Ethics oversight	All procedures were approved by the Stockholm South Ethical review board (Dnr. 06088-2020)

Note that full information on the approval of the study protocol must also be provided in the manuscript.

Flow Cytometry

Plots

Confirm that:

- [x] The axis labels state the marker and fluorochrome used (e.g. CD4-FITC).
- [x] The axis scales are clearly visible. Include numbers along axes only for bottom left plot of group (a 'group' is an analysis of identical markers).
- [x] All plots are contour plots with outliers or pseudocolor plots.
- [x] A numerical value for number of cells or percentage (with statistics) is provided.

Methodology

Sample preparation	Cells were prepared as described in method part. After cell sorting, cells were grown in RPMI 1640 media with 20% FBS and 1% penicillin/sptreptomycin for 3 days.
Instrument	FACSAria III sorter (BD Biosciences)
Software	FACSDiva Version 6.1.3
Cell population abundance	After filtering with the SSC-A/FSC-A signal, GFP-positive population for pEGFP-N1 transfected cells (positive control) were 78% on average, whereas for untreated cells (negative controls) were 0%.<br>GFP-positive population of ABEmax-VQR treated cells were about 7 % on average, and a total of 5x10^3 GFP-positive cells were collected to enrich the base editing frequency
Gating strategy	Untreated cells (negative control) and pEGFP-NI transfected cells (positive controls) were used for gating strategy.

- [x] Tick this box to confirm that a figure exemplifying the gating strategy is provided in the Supplementary Information.
